# The effect of flaxseed supplementation on sex hormone profile in adults: a systematic review and meta-analysis

**DOI:** 10.3389/fnut.2023.1222584

**Published:** 2023-10-20

**Authors:** Vali Musazadeh, Ahmad Nazari, Mohammad Natami, Zahra Hajhashemy, Kimia Sadat Kazemi, Fereshte Torabi, Amir Hossein Moridpour, Mahdi Vajdi, Gholamreza Askari

**Affiliations:** ^1^Student Research Committee, Tabriz University of Medical Sciences, Tabriz, Iran; ^2^School of Nutrition and Food Science, Tabriz University of Medical Sciences, Tabriz, Iran; ^3^Tehran University of Medical Sciences, Tehran, Iran; ^4^Department of Urology, Shahid Mohammadi Hospital, Hormozgan University of Medical Sciences, Bandar Abbas, Iran; ^5^Student Research Committee, Isfahan University of Medical Sciences, Isfahan, Iran; ^6^Faculty of Dentistry, Shahid Beheshti University of Medical Sciences, Tehran, Iran; ^7^Faculty of Medicine, Mashhad University of Medical Sciences, Mashhad, Iran; ^8^Department of Community Nutrition, School of Nutrition and Food Science, Nutrition and Food Security Research Center, Isfahan University of Medical Sciences, Isfahan, Iran

**Keywords:** flaxseed, sex hormones, gender, systematic review, meta-analysis

## Abstract

Inconsistent data suggest that flaxseed supplementation may have a role in sex hormones. We aimed to carry out a systematic review and meta-analysis of randomized controlled trials (RCTs) investigating effects of flaxseed supplementation on sex hormone profile. PubMed, Scopus, Embase, Cochrane Library, Web of Science databases, and Google Scholar were searched up to March 2023. Standardized mean difference (SMD) was pooled using a random-effects model. Sensitivity analysis, heterogeneity, and publication bias were reported using standard methods. The quality of each study was evaluated with the revised Cochrane risk-of-bias tool for randomized trials, known as RoB 2. Finding from ten RCTs revealed that flaxseed supplementation had no significant alteration in follicle-stimulating hormone (FSH) (SMD: −0.11; 95% CI: −0.87, 0.66: *p* = 0.783), sex hormone-binding globulin (SHBG) (SMD: 0.35; 95% CI: −0.02, 0.72; *p* = 0.063), total testosterone (TT) levels (SMD: 0.17; 95% CI: −0.07, 0.41; *p* = 0.165), free androgen index (FAI) (SMD = 0.11, 95% CI: −0.61, 0.83; *p* = 0.759), and dehydroepiandrosterone sulfate (DHEAS) (SMD: 0.08, 95%CI: −0.55, 0.72, *p* = 0.794). Flaxseed supplementation had no significant effect on sex hormones in adults. Nevertheless, due to the limited included trials, this topic is still open and needs further studies in future RCTs.

## 1. Introduction

Sex hormones and their harmony have been considered as an important issue, due to their interactions with body tissues. Estrogen and progesterone are the main female sex hormones that their production and secretion are triggered by pituitary hormones including follicle-stimulating hormone (FSH) and luteinizing hormone (LH) (1). Additionally, LH is the main regulatory hormone for testosterone in men (2). Besides the regulation of the reproductive system and maturation, these sex hormones have other critical roles in health status. Such that, estrogen protectively influences bone mass (3), immune system (4), and cardiovascular system (5). Muscle mass is also strengthened by androgens (6). Nevertheless, disharmony in sex hormones is involved in the incidence of several diseases including osteoarthritis (7), obesity, metabolic syndrome, polycystic ovary syndrome (PCOS), cardiovascular disease (CVD) (8), and different types of cancers (9, 10). Therefore, the prevention and management of disharmony in sex hormones are very worthful.

The synthesis and metabolism of sex hormones are influenced by various factors including genetics, lifestyle, dietary intake, physical activity and environmental factors (11, 12). Based on evidence, flaxseed might influence the sex hormone levels due to its phytoestrogens content such as lignans. The main lignan content of flaxseed is secoisolariciresinol diglycoside which is converted to the mammalian lignans enterolactone and enterodiol by intestinal bacteria (13). Due to similar structure of lignans to sex hormones, they could inhibit the aromatase activity and elevate the sex hormone-binding globulin (SHBG) synthesis in adipose tissues and the liver. Lignans could also bind to testosterone and increase its excretion in bile. Moreover, it has anti-cancer properties due to its inhabitation effect on cell proliferation and mammary tumor incidence (14, 15).

Although the lignan contents of flaxseed could regulate the sex hormones and consequently influence the incidence of related disease, the exact effect of flaxseed supplementation on sex hormones was not clear. Previous studies suggested that the consumption of flaxseed could alter the metabolism of estrogen in postmenopausal women (16). They illustrated that flaxseed supplementation could change serum levels of only some sex hormones (17) or even influence the metabolism and urinary levels of some estrogen metabolites (18). Hutchins et al. (19) reported that consumption of 5 or 10 g/day ground flaxseed for 7 weeks resulted in reduced *estradiol* levels in postmenopausal women. Moreover, a clinical trial illustrated the favorable effect of flaxseed on sex hormones, only in overweight and obese women (20). Haidari et al. (21) also found that supplementation with 3 g/day of flaxseed for a period of 12 weeks did not result in any significant reduction in serum testosterone or SHBG levels in patients with PCOS. Regarding the controversial findings of previous studies, we aimed to examine the probable effect of flaxseed intake on sex hormones such as FSH, SHBG, free androgen index (FAI), total testosterone (TT), and dehydroepiandrosterone sulfate (DHEAS) through a comprehensive systematic review and meta-analysis of randomized clinical trials (RCTs).

## 2. Method

The current meta-analysis was provided according to the PRISMA guideline (22). The protocol of the present study has been approved by the ethics committee of Isfahan University of Medical Sciences (identifier: IR.MUI.RESEARCH. REC.1402.031 and grant number: 140215).

### 2.1. Search strategy

A comprehensive systematic search was applied on Web of Science, Google Scholar, EMBASE, PubMed, and Scopus up to March 2023 without any restriction in language or publication year. Additionally, we checked the reference list of related articles to avoid missing the eligible studies. The details of keywords and search strategy in each database were provided in Supplementary Table S1.

### 2.2. Inclusion and exclusion criteria

Studies were eligible to be included if met the following requirements: (1) had randomized clinical controlled trial design; (2) investigated the influence of flaxseed on sex hormones or their binding proteins including [total testosterone, SHBG, follicle-stimulating hormone (FSH), free androgen index (FAI) and dehydroepiandrosterone Sulfate (DHEAS)]; (3) performed on men and women 18 years or older; (4) reported the change values of the mentioned variables or their values before and after intervention in both control and in treatment groups. Nevertheless, we excluded studies if: (1) used the combination of flaxseed with other substances or exercise; (2) had not control group; (3) did not apply random allocation; (4) did not report changes of the interested variables or their values before and after the intervention; (5) investigated the pregnant women, and children; and (6) gray literatures, patents, dissertations.

### 2.3. Data extraction

Two investigators independently performed data extraction and the principle researcher supervised this process (G.A). The necessary information was extracted including the duration, location, design and publication year of included studies, first author’s last name, the mean age, health status, and the number of included subjects, flaxseed dosage, and the mean ± standard deviation changes of sex hormones or their values before and after intervention in both groups. In studies with insufficient data, authors were requested to send more information by email.

### 2.4. Quality assessment

Two authors (VM and MV) independently evaluated the risk of bias for each study with RoB 2. The assessment focused on 5 different domains of each study: allocation concealment, random sequence generation, selective reporting, blinding of outcome assessment, and incomplete outcome data. For each section, algorithms assessed the potential bias (low risk, unclear risk, or high risk) (23).

### 2.5. Statistical analysis

Using the mean ± standard deviation (SD) changes of sex hormones in intervention and control groups, standardized mean difference (SMD) and 95% confidence intervals were estimated as the overall estimates (24). The Cochran’s Q test and inconsistency index (I-squared) were used to determine the between-study heterogeneity. Such that, *I*^2^ ≥ 75% and *p*-value of Q statistic <0.1 were defined as high between-study heterogeneity (25). In cases with significant between-study heterogeneity, subgroup analysis was applied to find the source of heterogeneity. Additionally, meta-regression was performed for continuous variables. Using the sensitivity analysis, the individual effect of each study on the overall effect size was examined. Through the use of funnel plots, Begg’s and Egger’s tests, we assessed the publication bias (26). All analyses were conducted through the use of Stata Statistical Software version 14 (Stata Corp, College Station, TX, United States).

## 3. Results

### 3.1. Selection and characteristics of studies

**Figure 1:**
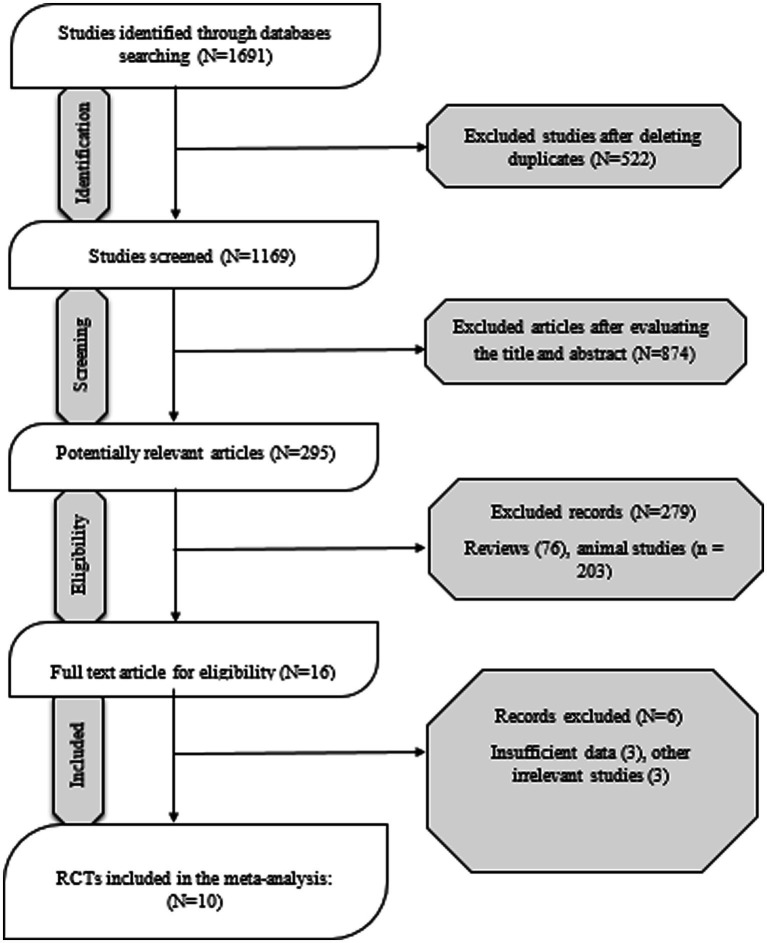
Flow diagram of study selection.

**Figure 2:**
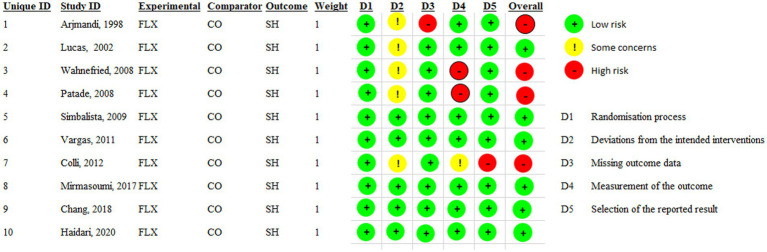
Risks of bias assessed by RoB2 for each included study (*n* = 10). FLX, Flaxseed; CO, Control; SH, Sex hormone.

A total of 1,691 articles were found through the initial literature search of PubMed, Embase, Cochrane, and Web of Science databases, of which 522 were duplicates, and 874 articles were excluded through screening titles and abstracts. Finally, 10 out 16 RCTs were included in the meta-analysis. The systematic review literature screening flow chart is shown in Figure 1. The characteristics of the included RCTs are outlined in Table 1. All included studies were conducted between 1998 and 2020. Of the 10 included studies, five of them were conducted in USA and two in Iran, two in Brazil, and one in Canada. The intervention duration of the studies ranged from 4.5 to 24 weeks; the mean age of the 49 years; and patient and control sample sizes ranged from 34 to 81. The risk of bias was assessed as shown in Figure 2. According to the RoB2, six of ten studies had high quality.

**Table 1:** Study characteristics of included studies.

Author, year	Design	Participants, *n*	Health condition	Age, year	Intervention		Duration (week)
					Treatment group	Control group	
Arjmandi, US, 1998	RA/DB/crossover	F: 34	Postmenopausal women	Int: 55.44, Con: 57.54	38,000 mg/day whole flaxseed	Sunflower seed	6
Lucas, USA, 2002	RA/DB/parallel	F: 36 Int: 20, Con: 16	Postmenopausal women	Int: 54, Con: 55	40,000 mg/day ground whole flaxseed	Wheat	12
Wahnefried, USA, 2008	RA/SB/parallel	M: 81 Int: 40, Con: 41 M: 80 Int: 40, Con: 40	Prostate Cancer	Int: 60.2, Con: 58.2 Int: 59.3, Con: 59.2	30,000 mg/day flaxseed-supplemented diet, 30,000 mg/day flaxseed-supplemented diet+ low-fat diet	Usual diet, low-fat diet	4.5
Patade, USA, 2008	RA/SB/parallel	F: 26 Int: 17, Con: 9	Postmenopausal women	47-63	30,000 mg/day flaxseed	Control	12
Simbalista, Brazil, 2009	RA/DB/parallel	F: 38 Int: 20, Con: 18	Postmenopausal women	Int: 52, Con: 52.7	25,000 mg/day ground flaxseed	Wheat bran	12
Vargas, USA, 2011	RA/DB/parallel	F: 34 Int: 17, Con: 17	PCOS	Int: 29.4, Con: 28.9	3,500 mg/day flaxseed oil (capsule)	Soybean oil	6
Colli, Brazil, 2012	RA/parallel	F: 53 Int: 28, Con: 25 F: 47 Int: 22, Con: 25	Menopausal	Int: 53.57, Con: 56.57 Int: 54.16, Con: 56.57	1,000 mg/d flaxseed Extract (lignan) 90,000 mg/d flaxseed meal	Collagen	24
Mirmasoumi, Iran, 2017	RA/DB/parallel	F: 60 Int: 30, Con: 30	PCOS	Int: 28.4, Con: 27	1000 mg/day flaxseed oil (capsule)	Liquid paraffin	12
Chang, Canada, 2018	RA/SB/parallel	F: 99 Int: 48, Con: 51	Postmenopausal women	60	15,000 mg/day ground flaxseed	Usual diet	7
Haidari, Iran, 2020	RA/parallel	F: 41 Int: 21, Con: 20	PCOS	Int: 27.21, Con: 26.13	30,000 mg/day brown milled flaxseed powder + lifestyle modification	Lifestyle modification	12

### 3.2. Effects of flaxseed on FSH

**Figure 3:**
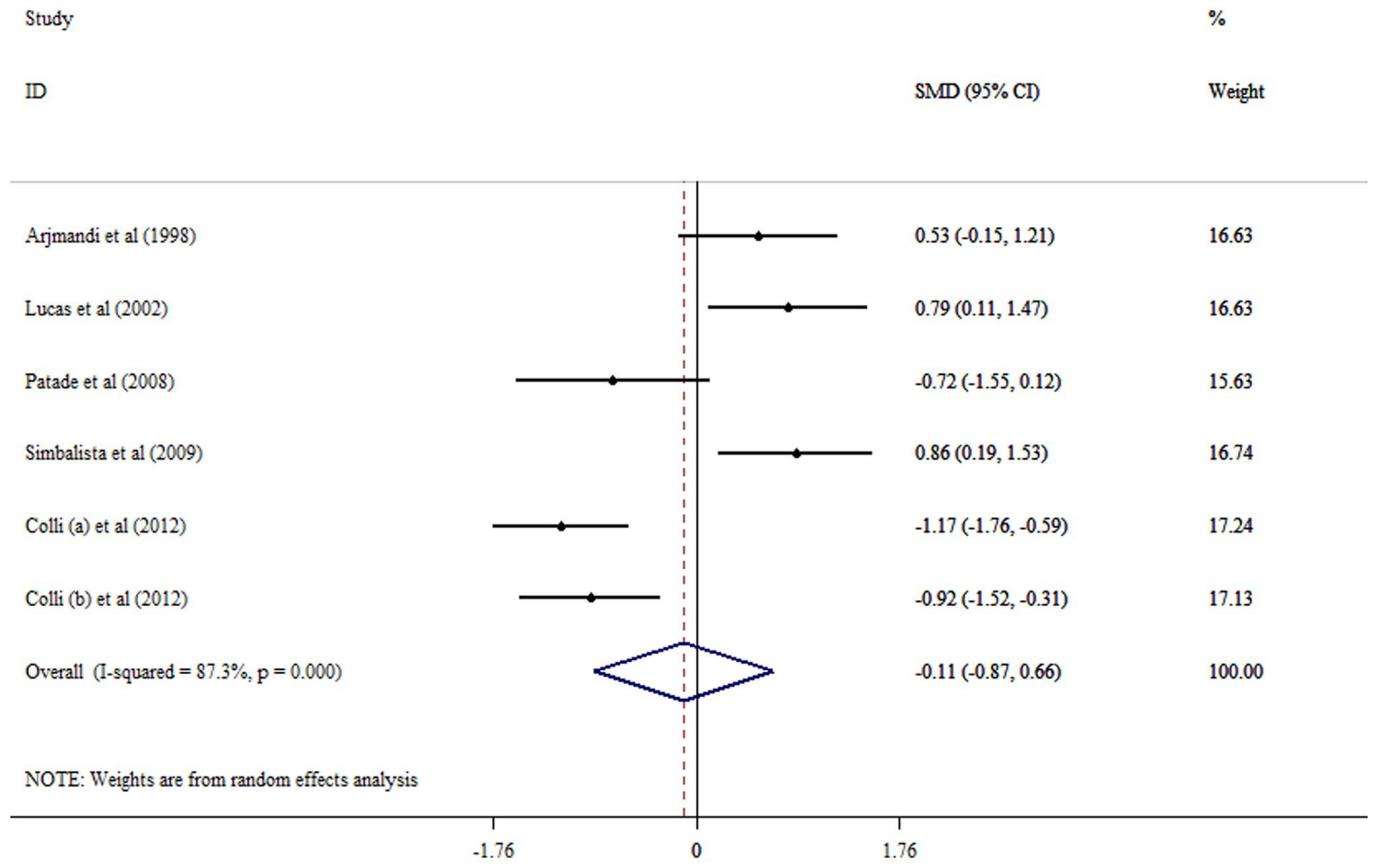
Forest plot detailing mean difference and 95% confidence intervals (CIs) the effects of flaxseed supplementation on FSH levels.

The pooled results showed that flaxseed supplementation no significantly decreased FSH (SMD: −0.11; 95% CI: −0.87, 0.66: *p* = 0.783, *I*^2^ = 87.3%, *p* < 0.001) (Figure 3). Flaxseed supplementation in studies with an intervention duration of ≥12 weeks contributes to a more significant reduction in FSH level (Table 2). Removing an individual study at a time by sensitivity analysis did not affect the results. Begg’s test revealed no significant in identifying small-study effects (*p* = 0.452).

**Table 2:** Subgroup analyses for the effects of Flaxseed supplementation on sexual hormone.

	No	No of participants	SMD (95% CI)^a^	P-within^b^	*I*^2^ (%)^c^	P-heterogeneity^d^
**Flaxseed on FSH**						
Overall	6	209	−0.11 (−0.87, 0.66)	0.783	87.3	<0.001
**Duration (week)**						
<12	4	134	0.40 (−0.25, 1.05)	0.227	70.0	0.019
≥12	2	75	−1.05 (−1.47, −063)	<0.001	0.0	0.552
**Flaxseed on SHBG**						
Overall	7	431	0.35 (−0.02, 0.72)	0.063	71.3	0.002
**Age (year)**						
≤50	3	135	0.38 (0.04, 0.72)	0.027	0.0	0.878
>50	4	296	0.36 (−0.26, 0.97)	0.261	85.0	<0.001
**Gender**						
Men	2	161	0.42 (−0.41, 1.24)	0.321	85.3	0.009
Women	5	270	0.32 (−0.14, 0.78)	0.169	69.4	0.011
**Duration (week)**						
<12	4	294	0.20 (−0.33, 0.72)	0.464	79.5	0.002
≥12	3	137	0.57 (0.18, 0.95)	0.004	18.0	0.295
**Study population**						
PCOS	3	135	0.38 (0.04, 0.72)	0.027	0.0	0.878
Prostate cancer	2	161	0.42 (−0.41, 1.24)	0.321	85.3	0.009
Post-menopausal	2	135	0.32 (−0.98, 1.63)	0.627	90.5	<0.001
**Flaxseed on total testosterone**						
Overall	6	395	0.17 (−0.07, 0.41)	0.165	27.7	0.227
**Age (year)**						
≤50	3	135	0.02 (−0.32, 0.36)	0.906	0.0	0.980
>50	3	260	0.27 (−0.15, 0.69)	0.205	65.1	0.057
**Gender**						
Men	2	161	0.48 (0.17, 0.79)	0.003	0.0	0.600
Women	4	234	−0.04 (−0.30, 0.22)	0.906	0.0	0.956
**Duration (week)**						
<12	4	294	0.24 (−0.10, 0.57)	0.464	49.5	0.115
≥12	2	101	0.00 (−0.39, 0.39)	0.004	0.0	0.998
**Study population**						
PCOS	3	135	0.02 (−0.32, 0.36)	0.906	0.0	0.980
Prostate cancer	2	161	0.48 (0.17, 0.79)	0.003	0.0	0.600
Post-menopausal	1	99	−0.12 (−0.51, 0.27)	0.551	–	–
**Flaxseed on FAI**						
Overall	4	262	0.11 (−0.61, 0.83)	0.759	87.6	<0.001
**Gender**						
Men	2	161	0.59 (−0.10, 1.29)	0.094	79.0	0.029
Women	2	101	−0.41 (−1.65, 0.82)	0.514	88.7	0.003
**Study population**						
PCOS	2	101	−0.41 (−1.65, 0.82)	0.514	88.7	0.003
Prostate cancer	2	161	0.59 (−0.10, 1.29)	0.094	79.0	0.029

SMD, standardized mean difference; CI, confidence interval.

a Obtained from the Random-effects model.

b Refers to the mean (95% CI).

c Inconsistency, percentage of variation across studies due to heterogeneity.

d Obtained from the Q-test.

### 3.3. Effect of flaxseed on SHBG

**Figure 4:**
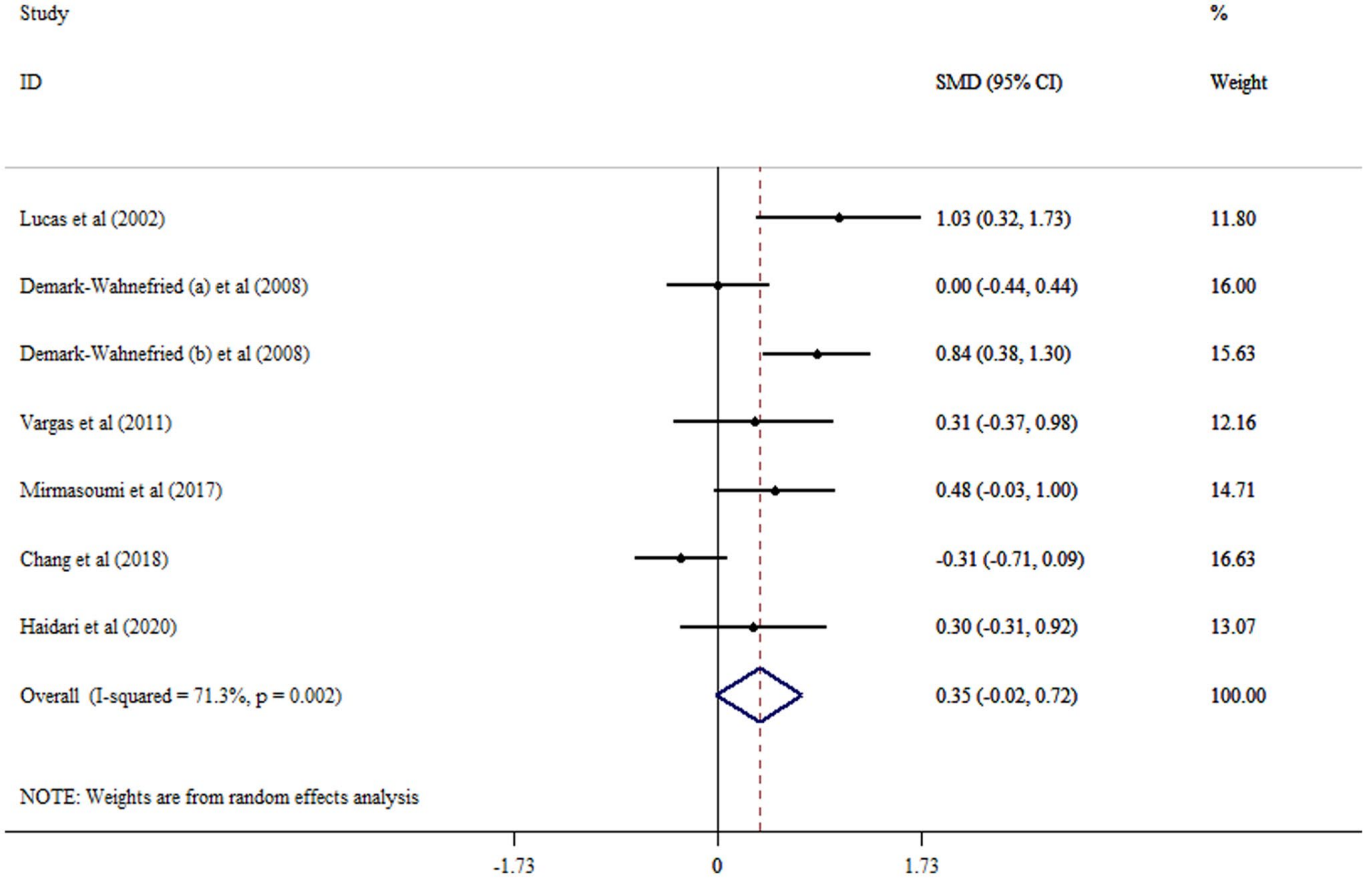
Forest plot detailing mean difference and 95% confidence intervals (CIs) the effects of flaxseed supplementation on SHBG levels.

Meta-analysis of data from six RCTs with seven arms revealed no significant alteration in SHBG following flaxseed supplementation (SMD: 0.35; 95% CI: −0.02, 0.72; *p* = 0.063, *I*^2^ = 71.3%, *p* = 0.002) (Figure 4). Subgroup analysis indicated that flaxseed supplementation in patients with PCOS, an intervention duration of ≥12 weeks and mean age of ≤50 years had a significant effect in increasing SHBG (Table 2). By removing Chang et al. study, the non-significant effect of flaxseed on SHBG levels became significant (SMD: 0.47; 95% CI: 0.16, 0.79; *p* < 0.05). The result of Begg’s tests was not significant in identifying small-study effects (*p* = 0.207).

### 3.4. Effect of flaxseed on TT

**Figure 5:**
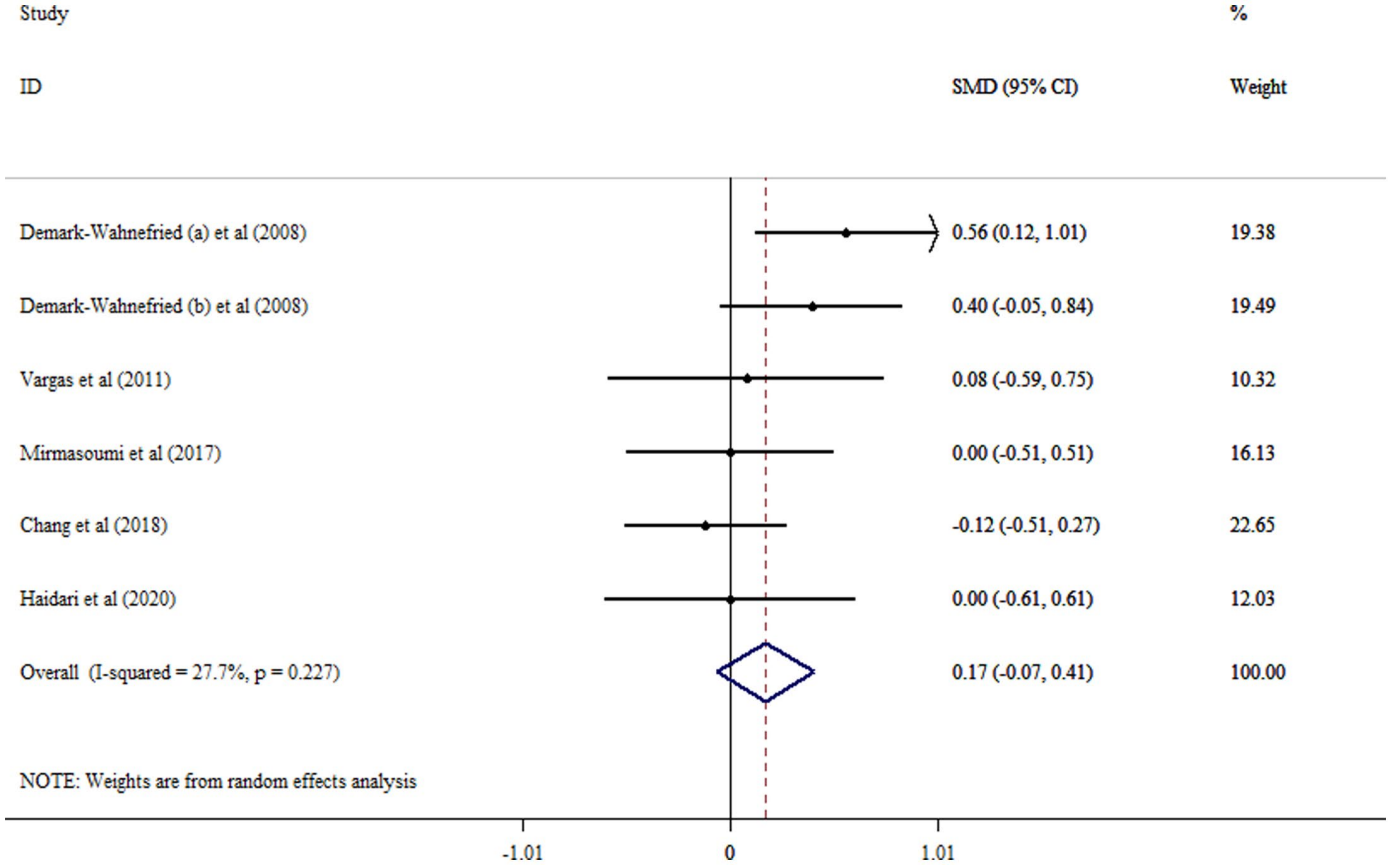
Forest plot detailing mean difference and 95% confidence intervals (CIs) the effects of flaxseed supplementation on TT levels.

The results of the meta-analysis indicated that flaxseed did not significantly increase TT levels (SMD: 0.17; 95% CI: −0.07, 0.41; *p* = 0.165, *I*^2^ = 27.7%, *p* = 0.227) (Figure 5). Performing subgroup analysis revealed that the effects of flaxseed on TT levels in men with prostate cancer were more robust than the entire sample (Table 2). By removing Chang et al. study, the non-significant effect of flaxseed on TT levels became significant (SMD: 0.26; 95% CI: 0.31, 0.49; *p* < 0.05). There were no significant small-study effects with using Begg’s test (*p* = 0.296).

### 3.5. Effect of flaxseed on FAI

**Figure 6:**
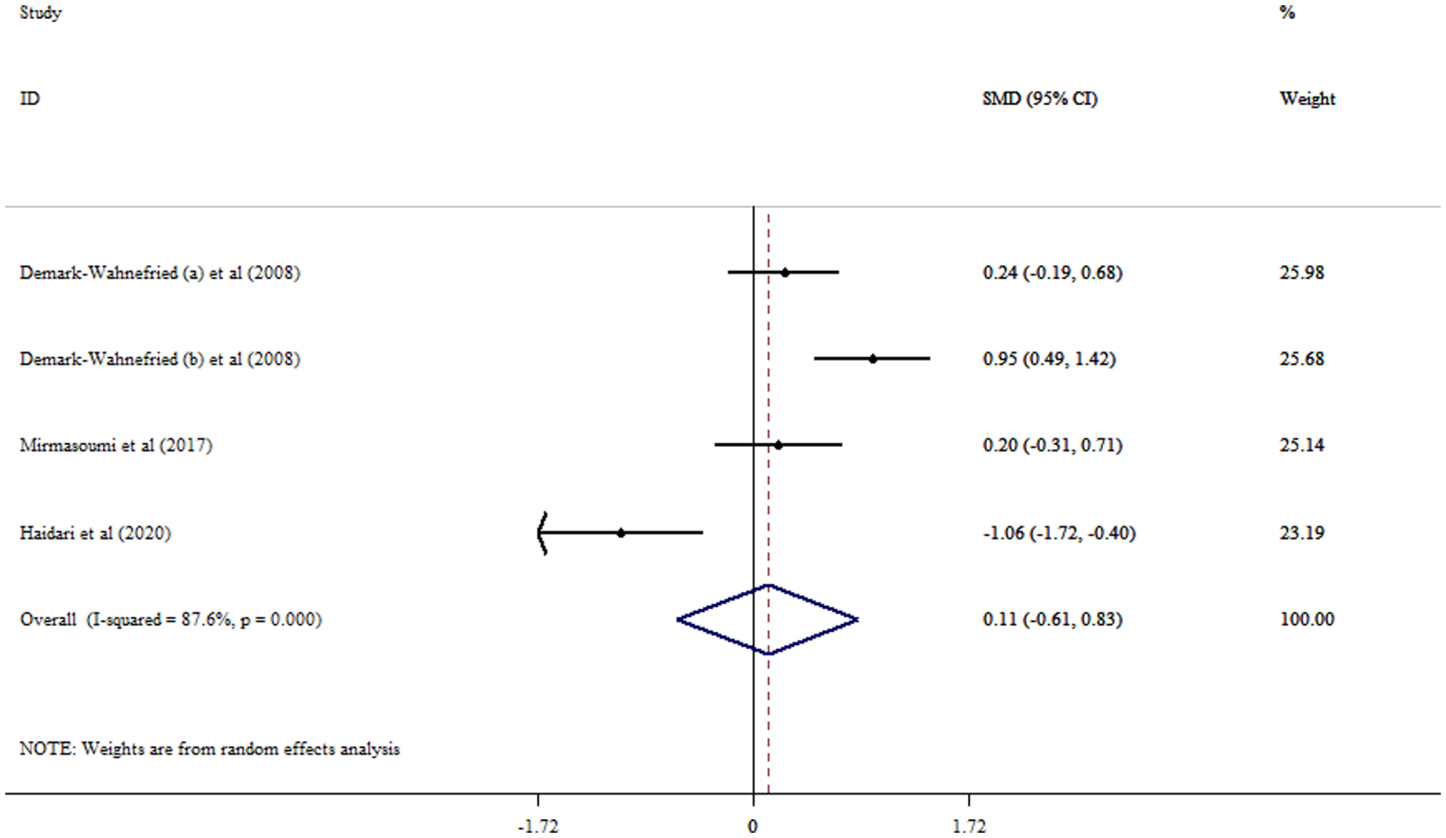
Forest plot detailing mean difference and 95% confidence intervals (CIs) the effects of flaxseed supplementation on FAI levels.

Results did not show any meaningful effect of flaxseed supplementation on FAI (SMD = 0.11, 95% CI: −0.61, 0.83; *p* = 0.759, *I*^2^ = 87.6%, *p* < 0.001) (Figure 6). No significant difference in overall effect size was shown after removing each study using sensitivity analysis.

### 3.6. Effect of flaxseed on DHEAS

**Figure 7:**
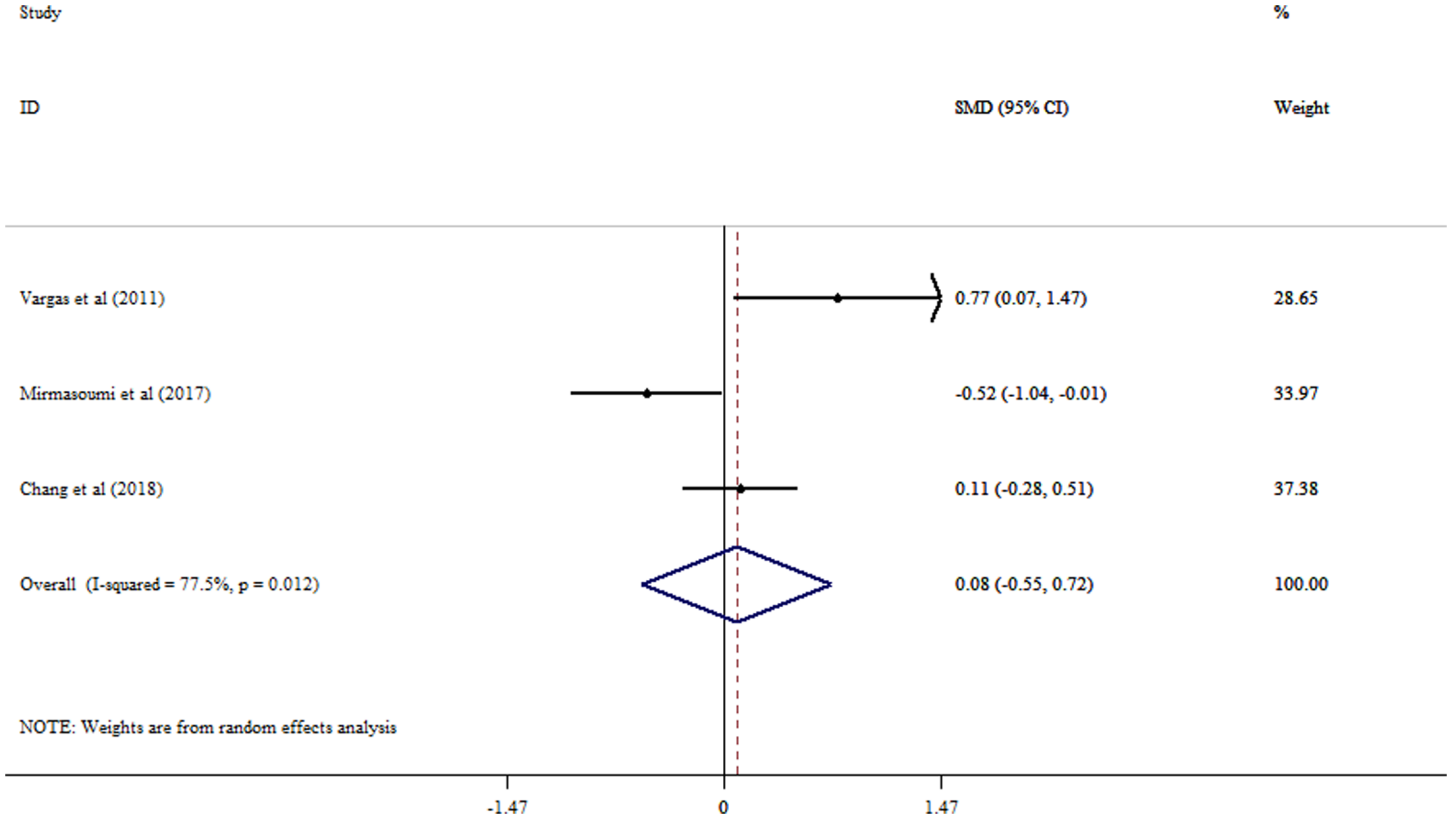
Forest plot detailing mean difference and 95% confidence intervals (CIs) the effects of flaxseed supplementation on DHEAS levels.

Flaxseed supplementation led to no significant increase in DHEAS (SMD: 0.08, 95%CI: −0.55, 0.72, *p* = 0.794, *I*^2^ = 77.5%, *p* = 0.012) (Figure 7).

## 4. Discussion

In the present systematic review and meta-analysis, we summarized the available data from 10 trials which investigated the effect of flaxseed supplementation on sex hormones. This study to the best of our knowledge is the first in this field of research. The meta-analysis did not show a significant effect of flaxseed on FSH, FAI, DHEAS, TT, and SHBG levels in comparison with control group in adults. However, subgroup analyses showed that flaxseed supplementation in subjects with ≤50 years old, an intervention duration of ≥12 weeks and with PCOS significantly increased SHBG. In addition, we observed an increase in TT following flaxseed supplementation in subgroup of men, and subjects with prostate cancer. Results from most trials in this area are in line with our study. In terms of estrogenic effects of flaxseed, it did not change FSH, FAI levels and contradicted previous results that flaxseed has estrogenic properties (16, 18). It should be taken into account that few RCTs have examined the effect of flaxseed supplementation on sex hormones. Consequently, further RCTs are required to reach a firm conclusion about the effect of flaxseed in different durations and doses on sex hormones.

The effects of dietary components on circulating sex hormone levels are potentially of great importance for the prevention of hormone-associated complaints. The endocrine system is a complex network of hormones and glands that decline with age includes a reduction in testosterone levels of 0.5–1% per year in men, and of estrogen in women, that initiates around 30 years of age (27, 28). The decline in testosterone levels in men is related to loss of muscle mass and strength, and moreover testosterone/dihydrotestosterone supplementation can increase muscle strength (29). Flaxseed is a rich source of several biologically active compounds such as plant lignin secoisolariciresinol diglucoside, which is metabolized to the mammalian lignans enterolactone and enterodiol by intestinal bacteria (13). These products are basically similar to endogenous sex hormones and have been revealed to exert weak hormonal properties and prevent carcinogenesis in animal studies (30, 31). For at least two decades, flaxseed, as well as lignans has been examined for their capability has been investigated for their capability to prevention of hormone-related cancers such as breast cancer (30, 32–34). Moreover, lignan has been revealed to decrease testosterone by binding it to enterohepatic circulation and 5α-reductase, the enzyme that converts testosterone to dihydrotestosterone (35, 36).

Morton et al. (37) reported that high lignan diets may be protective against prostate cancer, which is related to high androgen levels. Additionally, lignans have been reported to stimulate SHBG synthesis in the liver and interact with SHBG to change biological activity of circulating androgens and estrogens (38, 39). However, we cannot conclude that flaxseed consumption imposed a significant effect on FSH, FAI, and DHEAS in adult subjects. This might be due to the inconsistencies between the included trials, such as (a) different duration of the intervention period; (b) form and dose of flaxseed supplements used in the trials. In included trials, heterogeneity exists with regard to the role of flaxseed supplementation in lowering sex hormones levels. One potential source of heterogeneity was the intervention duration; where a short period may not be adequate to elicit significant alterations in sex hormones levels. It is also possible that an effect was actually existent but was unobserved in the current study due to the small sample sizes of the included trials, which resulted in a low statistical power for finding of significant results, and obviously represents a viable avenue for future studies.

The mechanism by which flaxseed supplementation could affect sex hormones is, currently, not well understood. Evidence from RCT by Chang et al. (17), provides some support for flaxseed’s role in changing estrogen metabolism rather than preventing estrogen synthesis. It has been suggested that flaxseed may change the profile of estrogen metabolites by changing the activity of cytochrome P450 enzymes responsible for estrogen hydroxylation (40, 41). Moreover, lignans have been proposed to exert anticancer effects by competing with estrogens for binding to estrogen receptors, resulting in changed estrogen-sensitive gene expression, and consequently, reduced cell proliferation and improved apoptosis (42). Another mechanism by which lignans appears to have a role in controlling sex hormones may be due to its interact with enzymes involved in hormone metabolism and synthesis to control relative levels of circulating sex hormones (43). *In vitro* studies have proposed that lignans may decline estrogen synthesis by impeding the aromatase enzyme responsible for altering androstenedione and testosterone to estrone and estradiol, respectively.

### 4.1. Strengths and limitations

This is the first systematic review and meta-analysis of RCTs examining the effect of flaxseed supplementation on sex hormones. The lack of publication bias as evidenced by Egger’s and Begg’s test propose that our result was reliable, since trials which could potentially change the overall results were not evident. Moreover, most of the included trials were designated as low risk of bias according to Cochrane risk of bias tool. However, there are some limitations that can be addressed in future studies. The number of RCTs that met the eligibility criteria for inclusion in the meta-analysis, as well as the number of men included in these trials, were limited. This made it challenging to accurately assess the clinical effectiveness of flaxseed on sex hormone. Included studies were conducted on participants with varying health conditions (PCOS, postmenopausal women, and prostate cancer), and only a small proportion of the participants were young and healthy. It was not possible to examine the effects of flaxseed on other sex hormones due to inadequate dataset. Studies about the effect of flaxseed supplementation on FSH, FAI, and DHEAS were very low. So, more studies are needed to confirm our findings about these hormones. The included studies involved participants with diverse health status. Another limitation is the difference in the form of flaxseed, the preparation method, and period of intervention. Moreover, the number of trials and included subjects were small.

## 5. Conclusion

In conclusion, we found no significant effect of flaxseed on sex hormones in adults. However, according to subgroup analyses flaxseed supplementation increased SHBG in subjects with ≤50 years old and with PCOS, and TT in men. Nevertheless, due to the discussed limitations of the included trials, this topic is still open and needs further studies in future RCTs.

## Data availability statement

The original contributions presented in the study are included in the article/Supplementary material, further inquiries can be directed to the corresponding authors.

## Ethics statement

The study protocol was approved and registered by the ethics committee of Isfahan University of Medical Sciences (identifier: IR.MUI.RESEARCH. REC.1402.031).

## Author contributions

VM and MV was responsible for designing and coordinating the study. KK, FT, VM, AM, and MV were responsible for data collection, data analysis, and data interpretation in the manuscript. MV and MN were responsible for the statistical work and for writing the manuscript. GA was responsible for reviewing the manuscript. All authors read and approved the final manuscript.

## References

[1] Haggstrom M. Reference ranges for estradiol, progesterone, luteinizing hormone and follicle-stimulating hormone during the menstrual cycle. WikiJournal Med. (2014) 1:1–5. doi: 10.15347/wjm/2014.001

[2] Ramaswamy S Weinbauer GF. Endocrine control of spermatogenesis: role of FSH and LH/testosterone. Spermatogenesis. (2014) 4:e996025. doi: 10.1080/21565562.2014.996025 26413400 PMC4581062

[3] Levin V Jiang X Kagan R. Estrogen therapy for osteoporosis in the modern era. Osteoporos Int. (2018) 29:1049–55. doi: 10.1007/s00198-018-4414-z, PMID: 29520604

[4] Khan D Ansar Ahmed S. The immune system is a natural target for estrogen action: opposing effects of estrogen in two prototypical autoimmune diseases. Front Immunol. (2016) 6:635. doi: 10.3389/fimmu.2015.00635 26779182 PMC4701921

[5] Knowlton A Lee A. Estrogen and the cardiovascular system. Pharmacol Ther. (2012) 135:54–70. doi: 10.1016/j.pharmthera.2012.03.007, PMID: 22484805 PMC5688223

[6] Bhasin S. Regulation of body composition by androgens. J Endocrinol Investig. (2003) 26:814–22. doi: 10.1007/BF03345230, PMID: 14964432

[7] Linn S Murtaugh B Casey E. Role of sex hormones in the development of osteoarthritis. PM&R. (2012) 4:S169–73. doi: 10.1016/j.pmrj.2012.01.013 22632696

[8] Guarner-Lans V Rubio-Ruiz ME Pérez-Torres I de MacCarthy GB. Relation of aging and sex hormones to metabolic syndrome and cardiovascular disease. Exp Gerontol. (2011) 46:517–23. doi: 10.1016/j.exger.2011.02.007, PMID: 21397002

[9] Demark-Wahnefried W Polascik TJ George SL Switzer BR Madden JF Ruffin MT . Flaxseed supplementation (not dietary fat restriction) reduces prostate cancer proliferation rates in men presurgery. Cancer Epidemiol Biomark Prev. (2008) 17:3577–87. doi: 10.1158/1055-9965.EPI-08-0008, PMID: 19064574 PMC2703189

[10] Folkerd EJ Dowsett M. Influence of sex hormones on cancer progression. J Clin Oncol. (2010) 28:4038–44. doi: 10.1200/JCO.2009.27.4290, PMID: 20644089

[11] Bjørnerem AS Straume B Midtby M Fønnebø V Sundsfjord J Svartberg J . Endogenous sex hormones in relation to age, sex, lifestyle factors, and chronic diseases in a general population: the Tromsø study. J Clin Endocrinol Metabol. (2004) 89:6039–47. doi: 10.1210/jc.2004-0735 15579756

[12] Wiggs AG Chandler JK Aktas A Sumner SJ Stewart DA . The effects of diet and exercise on endogenous estrogens and subsequent breast cancer risk in postmenopausal women. Front Endocrinol. (2021) 12:732255. doi: 10.3389/fendo.2021.732255, PMID: 34616366 PMC8489575

[13] Thompson LU Robb P Serraino M Cheung F. Mammalian lignan production from various foods. Nutr Cancer. (1991) 16:43–52. doi: 10.1080/01635589109514139 1656395

[14] Frische EJ Hutchins AM Martini MC Thomas W Slavin JL. Effect of flaxseed and wheat bran on serum hormones and lignan excretion in premenopausal women. J Am Coll Nutr. (1983) 22:550–54. doi: 10.1080/07315724.2003.10719335 14684762

[15] Chen J Tan KP Ward WE Thompson LU. Exposure to flaxseed or its purified lignan during suckling inhibits chemically induced rat mammary tumorigenesis. Exp Biol Med. (2003) 228:951–8. doi: 10.1177/153537020322800811, PMID: 12968067

[16] Brooks JD Ward WE Lewis JE Hilditch J Nickell L Wong E . Supplementation with flaxseed alters estrogen metabolism in postmenopausal women to a greater extent than does supplementation with an equal amount of soy. Am J Clin Nutr. (2004) 79:318–25. doi: 10.1093/ajcn/79.2.318, PMID: 14749240

[17] Chang VC Cotterchio M Boucher BA Jenkins DJA Mirea L McCann SE . Effect of dietary flaxseed intake on circulating sex hormone levels among postmenopausal women: a randomized controlled intervention trial. Nutr Cancer. (2019) 71:385–98. doi: 10.1080/01635581.2018.1516789, PMID: 30375890

[18] Sturgeon SR Volpe SL Puleo E Bertone-Johnson ER Heersink J Sabelawski S . Effect of flaxseed consumption on urinary levels of estrogen metabolites in postmenopausal women. Nutr Cancer. (2010) 62:175–80. doi: 10.1080/01635580903305342, PMID: 20099191

[19] Hutchins AM Martini MC Olson BA Thomas W Slavin JL. Flaxseed consumption influences endogenous hormone concentrations in postmenopausal women. Nutr Cancer. (2001) 39:58–65. doi: 10.1207/S15327914nc391_8, PMID: 11588903

[20] Sturgeon SR Heersink JL Volpe SL Bertone-Johnson ER Puleo E Stanczyk FZ . Effect of dietary flaxseed on serum levels of estrogens and androgens in postmenopausal women. Nutr Cancer. (2008) 60:612–8. doi: 10.1080/01635580801971864, PMID: 18791924

[21] Haidari F Banaei-Jahromi N Zakerkish M Ahmadi K. The effects of flaxseed supplementation on metabolic status in women with polycystic ovary syndrome: a randomized open-labeled controlled clinical trial. Nutr J. (2020) 19:8:1–11. doi: 10.1186/s12937-020-0524-5 31980022 PMC6982376

[22] Moher D Shamseer L Clarke M Ghersi D Liberati A Petticrew M . Preferred reporting items for systematic review and meta-analysis protocols (PRISMA-P) 2015 statement. Syst Rev. (2015) 4:1–9. doi: 10.1186/2046-4053-4-1, PMID: 25554246 PMC4320440

[23] Sterne JA Savović J Page MJ Elbers RG Blencowe NS Boutron I . RoB 2: a revised tool for assessing risk of bias in randomised trials. Br Med J. (2019) 366:l4898. doi: 10.1136/bmj.l4898 31462531

[24] DerSimonian R Kacker R. Random-effects model for meta-analysis of clinical trials: an update. Contemp Clin Trials. (2007) 28:105–14. doi: 10.1016/j.cct.2006.04.004, PMID: 16807131

[25] Higgins JP Thompson SG Deeks JJ Altman DG. Measuring inconsistency in meta-analyses. BMJ. (2003) 327:557–60. doi: 10.1136/bmj.327.7414.557, PMID: 12958120 PMC192859

[26] Lin L Chu H. Quantifying publication bias in meta‐analysis. Biometrics. (2018) 74:785–94. doi: 10.1111/biom.12817 29141096 PMC5953768

[27] Baumgartner RN Waters DL Gallagher D Morley JE Garry PJ. Predictors of skeletal muscle mass in elderly men and women. Mech Ageing Dev. (1999) 107:123–36. doi: 10.1016/S0047-6374(98)00130-4, PMID: 10220041

[28] Maggio M Lauretani F Ceda GP. Sex hormones and sarcopenia in older persons. Curr Opin Clin Nutr Metab Care. (2013) 16:3–13. doi: 10.1097/MCO.0b013e32835b6044 23222704

[29] Ottenbacher KJ Ottenbacher ME Ottenbacher AJ Acha AA Ostir GV. Androgen treatment and muscle strength in elderly men: a meta-analysis. J Am Geriatr Soc. (2006) 54:1666–73. doi: 10.1111/j.1532-5415.2006.00938.x, PMID: 17087692 PMC1752197

[30] Adlercreutz H. Lignans and human health. Crit Rev Clin Lab Sci. (2007) 44:483–525. doi: 10.1080/10408360701612942 17943494

[31] Thompson LU. 9Experimental studies on lignans and cancer. Baillieres Clin Endocrinol Metab. (1998) 12:691–705. doi: 10.1016/S0950-351X(98)80011-6, PMID: 10384820

[32] Touré A Xueming X. Flaxseed lignans: source, biosynthesis, metabolism, antioxidant activity, bio-active components, and health benefits. Compr Rev Food Sci Food Saf. (2010) 9:261–9. doi: 10.1111/j.1541-4337.2009.00105.x, PMID: 33467817

[33] Zaineddin AK Vrieling A Buck K Becker S Linseisen J Flesch-Janys D . Serum enterolactone and postmenopausal breast cancer risk by estrogen, progesterone and herceptin 2 receptor status. Int J Cancer. (2012) 130:1401–10. doi: 10.1002/ijc.26157, PMID: 21544804

[34] Buck K Zaineddin AK Vrieling A Linseisen J Chang-Claude J. Meta-analyses of lignans and enterolignans in relation to breast cancer risk. Am J Clin Nutr. (2010) 92:141–53. doi: 10.3945/ajcn.2009.28573, PMID: 20463043

[35] Denis L Morton M Griffiths K. Diet and its preventive role in prostatic disease. Eur Urol. (1999) 35:377–87. doi: 10.1159/000019912, PMID: 10325492

[36] McCann MJ Gill CIR McGlynn H Rowland IR. Role of mammalian lignans in the prevention and treatment of prostate cancer. Nutr Cancer. (2005) 52:1–14. doi: 10.1207/s15327914nc5201_1, PMID: 16090998

[37] Morton M Chan PSF Cheng C Blacklock N Matos-Ferreira A Abranches-Monteiro L . Lignans and isoflavonoids in plasma and prostatic fluid in men: samples from Portugal, Hong Kong, and the United Kingdom. Prostate. (1997) 32:122–8. doi: 10.1002/(SICI)1097-0045(19970701)32:2<122::AID-PROS7>3.0.CO;2-O, PMID: 9215400

[38] Martin ME Haourigui M Pelissero C Benassayag C Nunez EA. Interactions between phytoestrogens and human sex steroid binding protein. Life Sci. (1995) 58:429–36. doi: 10.1016/0024-3205(95)02308-9, PMID: 8594308

[39] Schöttner M Ganßer D Spitelle G. Interaction of lignans with human sex hormone binding globulin (SHBG). Zeitschrift Naturforschung C. (1997) 52:834–43. doi: 10.1515/znc-1997-11-1218, PMID: 9463941

[40] Lord RS Bongiovanni B Bralley JA. Estrogen metabolism and the diet-cancer connection: rationale for assessing the ratio of urinary hydroxylated estrogen metabolites. Altern Med Rev. (2002) 7:112–29. PMID: 11991791

[41] McCann SE Wactawski-Wende J Kufel K Olson J Ovando B Kadlubar SN . Changes in 2-hydroxyestrone and 16α-hydroxyestrone metabolism with flaxseed consumption: modification by COMT and CYP1B1 genotype. Cancer Epidemiol Biomark Prev. (2007) 16:256–62. doi: 10.1158/1055-9965.EPI-06-0633, PMID: 17301257 PMC5613245

[42] Mason JK Thompson LU. Flaxseed and its lignan and oil components: can they play a role in reducing the risk of and improving the treatment of breast cancer? Appl Physiol Nutr Metab. (2014) 39:663–78. doi: 10.1139/apnm-2013-0420, PMID: 24869971

[43] Brooks JD Thompson LU. Mammalian lignans and genistein decrease the activities of aromatase and 17β-hydroxysteroid dehydrogenase in MCF-7 cells. J Steroid Biochem Mol Biol. (2005) 94:461–7. doi: 10.1016/j.jsbmb.2005.02.002, PMID: 15876411

