# Supplementary material for: The effect of flaxseed supplementation on sex hormone profile in adults: a systematic review and meta-analysis
Source: Front Nutr. 2023 Oct 20;10:1222584. doi: 10.3389/fnut.2023.1222584 (PMC10623424; doi:10.3389/fnut.2023.1222584)
Supplement: Supplementary file 1 [file Table_1.DOCX]

**Supplementary Table. 1.** The search strategy was developed using the following Mesh terms and keywords:

(Flax [MeSH] OR flaxseed [Title/Abstract] OR flaxseed [Title/Abstract] OR linseed [tiab] OR lignan [Title/Abstract] OR “whole flaxseed” [tiab] OR “ground flaxseed” [Title/Abstract] OR “flaxseed oil” [tiab] OR “Linum usitatissimum” [Title/Abstract] )AND ( “ovulation” [Mesh] OR “total testosterone” [Title/Abstract] OR “progesterone” [Title/Abstract] OR “sexual function” [Title/Abstract] OR “impotence/erectile/dysfunction” [ Title/Abstract] OR “sex hormone-binding globulin (SHBG) ” [ Title/Abstract] OR “Polycystic Ovary Syndrome” [ Title/Abstract] OR “Ovary Syndrome” [ Title/Abstract] OR “Polycystic Syndrome” [ Title/Abstract] OR “Polycystic Ovary” [ Title/Abstract] OR “Polycystic ovary disease” [ Title/Abstract] OR “Stein- Leventhal Syndrome” [ Title/Abstract] OR “Stein Leventhal Syndrome” [ Title/Abstract] OR “Syndrome, Stein-Leventhal” [ Title/Abstract] OR “Sclerocystic Ovarian Degeneration” [ Title/Abstract] OR “Ovarian Degeneration, Sclerocystic” [ Title/Abstract]) AND ("randomized controlled trial"[Publication Type] OR "controlled clinical trial"[Publication Type] OR "controlled clinical trial"[All Fields] OR randomized[Title/Abstract] OR randomized [Title/Abstract] OR placebo[Title/Abstract] OR "clinical trials as topic"[MeSH Terms] OR "cross-over studies"[MeSH Terms] OR "cross-over studies"[All Fields] OR "cross over studies"[All Fields] OR "Cross-over study"[All Fields] OR "Cross over study"[All Fields] OR "clinical trial"[Publication Type] NOT animals[All Fields] ).
